# Harsh Environmental-Tolerant and High-Performance Triboelectric Nanogenerator Based on Nanofiber/Microsphere Hybrid Membranes

**DOI:** 10.3390/ma16020562

**Published:** 2023-01-06

**Authors:** Dequan Sun, Ruirui Cao, Haoyi Wu, Xin Li, Haoran Yu, Lijin Guo

**Affiliations:** 1 School of Mechanical and Electrical Engineering, Zhengzhou Tourism College, Zhengzhou 451464, China; 2 Henan Key Laboratory of Photovoltaic Materials, Henan University, Kaifeng 475004, China; 3 School of Control Science and Engineering, Tiangong University, Tianjin 300387, China

**Keywords:** nanofiber/microsphere hybrid membrane, triboelectric nanogenerator, enhanced output performance, harsh environmental-tolerant

## Abstract

Triboelectric nanogenerator (TENG) can convert tiny mechanical energy into precious electrical energy. Constant improvements to the output performance of TENG is not only the driving force for its sustainable development, but also the key to expand its practical applicability in modern smart devices. However, most previous studies were conducted at room temperature, ignoring the influence of temperature on the output performance of TENG. Additionally, due to thermionic emission effect, the electrons transferred to a dielectric surface can be released into a vacuum after contact electrification. Therefore, TENG cannot maintain an effective electrical output under high-temperature conditions. Here, a series of high-temperature operatable flexible TENGs (HO-TENGs) based on nanofiber/microsphere hybrid membranes (FSHMs) was fabricated by electrospinning and electrospraying. The *V_oc_* of HO-TENG is 212 V, which is 2.33 times higher than that of control TENG. After 10,000 cycle stability tests, the HO-TENG shows excellent durability. Especially, this HO-TENG can maintain 77% electrical output at 70 °C compared to room temperature, showing excellent high-temperature operability. This study can not only provide a reference for the construction of advanced high-performance TENG, but also provide a certain experimental basis for efficient collection of mechanical energy in high-temperature environment and promote the application of TENG devices in harsh environments.

## 1. Introduction

Energy is one of the key factors for a country’s scientific and technological progress and social development [1,2,3]. At present, the traditional fossil fuels with limited reserves are still the main source of energy for human beings, but the burning of fossil fuels has caused serious environmental pollution and greenhouse effects [4,5,6,7]. With the rapid development of global industrialization, environmental pollution and energy shortages are two major problems facing the current world [8,9]. Therefore, searching for renewable energy with reduced carbon emissions, secure long-term energy supply, and less dependence on fossil fuels is mandatory for the sustainable development of the world [10,11].

In China’s National 14th Five-Year Plan and 2035 Vision Goal Outline, it is pointed out that we should promote the energy revolution, build a clean, low-carbon, safe and efficient modern energy system, and improve the energy supply and security capacity. At the same time, the Chinese Ministry of Science and Technology, China’s Ministry of Education, and the Chinese Academy of Science issued by “Nanotechnology” key special also explicitly proposed the use of energy conversion and storage in the nano-materials technology, nano energy and environmental technology as the key development direction and research task, strive to resolve environmental load, low energy efficiency, resource bottlenecks and other major common problems, and committed to the development of a series of environmental protection, low energy consumption, renewable green energy technology. From these, it is particularly important to explore new ways of harvesting energy, which has also received close attention in recent years [12]. There are various forms of mechanical energy in the daily environment, such as human motion energy, wind energy, water wave energy, etc., which have an abundance of reserves and come from a wide range of sources [13,14]. However, they are often ignored and cannot be utilized effectively due to their low energy density, low frequency and low utilization efficiency.

Recently, researchers have carried out a series of basic research and exploration in order to effectively recycle the wasted mechanical energy from the environment. A growing number of new generators with different energy conversion mechanisms have been developed, such as an electromagnetic generator, a piezoelectric generator, an electrostatic generator and a triboelectric nanogenerator (TENG) [15,16]. Due to the advantages of a simple structure, a low cost, a small size, being eco-friendly, and a high conversion efficiency [17,18,19,20,21], a TENG based on the coupling effect of contact electrification (CE) and electrostatic induction, has aroused strong concerns in the fields of nanoenergy, especially in the fields of micro-nano energy, blue energy and active sensing [20,21,22,23,24]. Therefore, continuous improvement of the TENG's output performance is not only the driving force and primary requirement for its development but is also crucial for expanding its practical applicability in modern smart devices [25,26,27]. It is well known that the electron is the dominant CE transferred charge identity, and the total surface charge output of TENG is reasonably interpreted as the direct result of the coupling of the electron thermionic emission rate, the charge transfer rate of CE and the contact area change rate between the two triboelectric materials [28,29,30,31]. At high temperatures, the electrostatic charge on the TENG surface can be released from the surface through electron thermionic emission and/or photon excitation [30,32]. Meanwhile, thermionic emission is not the only crucial element affecting the fundamental mechanism of CE [28,33], but also has an inevitable effect on almost all the triboelectric materials [30,33]. By reason of the thermionic emission effect, the electrons transferred to the surface of the triboelectric layer can be released into vacuum after CE, which inhibits the generation of a triboelectric charge and the output performance of the TENG. As a result, the TENG cannot maintain an effective electrical output performance at a high temperature, as it exhibits obvious thermal negative effects [32,34]. However, most of the previous studies were conducted in a single external environment [35], ignoring the influence of environmental instability (such as temperature) on the output performance of TENG devices. Recently, to improve the working temperature of TENG devices, researchers have gradually carried out some related research and explorations, but the electrical output performance of the fabricated TENG devices at high temperature is significantly lower than that at room temperature [36,37,38]. Recently, autonomous vehicles (driverless, autonomous, and robotic) that can sense the environment and navigate without human input based on artificial intelligence (AI) technology have proven to be innovative vehicles. Thereinto, the driving safety warning (DSEW) system is very important in the cruise of autonomous vehicles, which is a key technology proposed and rapidly developed in this field [39,40]. In fact, without a powerful and efficient sensor network, information cannot be provided. TENG can capture vibration/slide energy from a moving vehicle as a power source and a self-powered sensor for a DSEW system [40]. However, several limitations have previously existed with different types of TENG: (1) the supporting surface of the triboelectric material is prone to damage during contact and separation, and (2) most TENG devices are unable to maintain effective electrical output at high operating temperatures. Therefore, the application of a TENG at a high temperature is still severely limited, and further excavation and development of advanced triboelectric materials with wear resistance and high-temperature resistance is the key to solve this problem, which is also one of the most challenging tasks.

Previous studies confirmed that the single-electrode mode TENG (S-TENG) is an electrostatic system with inherent capacitance in nature, which can be equivalent to a circuit consisting of a series of capacitors [41,42]. Thereinto, when the external surface contacts the dielectric layer, the values of *V_oc_* and *Q_sc_* are both 0. When the external surface is far away from the dielectric layer, the *V_oc_* and *Q_sc_* of S-TENG can be expressed by the following equations [41,43],

(1) $$V_{oc}=-\sigma A/\left({2C}_{o}\right)$$

(2) $$Q_{sc}=-\sigma A/2$$

where *σ* is the electrostatic charge density generated on the surface of the dielectric layer, and *A* is the contact area of S-TENG in contact with triboelectric material. It can be seen from the above Equations (1) and (2) that the *V_oc_* and *Q_sc_* of S-TENG are positively correlated to the contact area *A* of the device. Therefore, by constructing micro/nanostructures on the contact surface and improving the effective contact area of CE, the electron exchange between the interface of the triboelectric materials can be effectively promoted, thus improving the triboelectric output performance of S-TENG devices [36,44,45,46]. However, additional complex manufacturing steps with high cost and poor wear resistance of micro/nanostructure are still the bottleneck for practical applications [46,47]. Therefore, without destroying its long-term durability, ease of manufacture and low cost, realizing the enhanced output performance of TENG is still the key to further promote its practical applications.

Herein, a series of harsh environmental-tolerant and high-performance TENGs (high-temperature operatable TENG, HO-TENG) based on nanofiber/microsphere hybrid membranes were prepared by electrospinning assisted with elecrospraying. This preparation technology has the advantages of simple preparation process, low cost and large-scale industrialization. The electrical output performance and durability of HO-TENGs were tested to verify the feasibility of this method to improve the output performance of the TENG devices and the effective operation of HO-TENG in a high-temperature environment. In conclusion, this study can not only provide a reference for the construction of advanced high-performance TENG, but also provide a certain experimental basis for the efficient collection of mechanical energy under high-temperature environment and promote the application of TENG devices in harsh environments.

## 2. Experimental Section

### 2.1. Materials

Poly(vinylidene fluoride-*co*-hexafluoropropylene) (PVDF-HFP) was purchased from the Arkema company. The solvents *N,N*-dimethylformamide (DMF, AR) and acetone (AR) were purchased from Sinopharm Chemical Reagent Co., Ltd. (Shanghai, China) and used as received. The conductive elastic fabric (SCN019) was purchased from Suzhou TEK Silver Fiber Technology Co., Ltd. (Suzhou, China).

### 2.2. Fabrication of Nanofiber and Nanofiber/Microsphere Hybrid Membranes

*Fabrication of nanofiber membrane (FM):* A certain mass (1.8 g, 3.0 g, 3.6 g, and 4.2 g) of PVDF-HFP was separately dissolved in DMF/acetone (60 wt%/40 wt%) mixture solution (13.2 g, 12.0 g, 11.4 g, and 10.8 g), and stirred at room temperature until completely dissolved (about 8 h, 250 rpm) to obtain the desired spinning solution. Then, the FM was fabricated by electrospinning and a rotating drum with a speed of 500 rpm was used as the receiver. The feed rate of the spinning solution was 1.0 mL/h with the relative humidity (38%) and at room temperature. The spinning voltage was about 12 kV, and the receiving distance was 20 cm.

*Fabrication of the nanofiber/microsphere hybrid membrane (FSHM):* The FSHM was fabricated on the electrospun nanofiber substrate collected above by elecrospraying technology. In brief, the FSHM was prepared by electrospinning assisted with elecrospraying. Thereinto, the solution used in the elecrospraying process was PVDF-HFP solution (DMF/acetone, 70 wt%/30 wt%) with a mass fraction of 16 wt%. The spinning voltage was about 13.50 kV and the receiving distance was 20 cm.

*Fabrication of the control sample:* The control sample was prepared by blade coating technology. The coating solution was PVDF-HFP solution (DMF/acetone, 60 wt%/40 wt%) with a mass fraction of 24 wt%. We scraped the above solution on the pre-cleaned glass pane and dried it at 80 °C for 40 min to obtain the PVDF-HFP membrane control sample.

### 2.3. Fabrication of Flexible HO-TENG

**Scheme 1:**
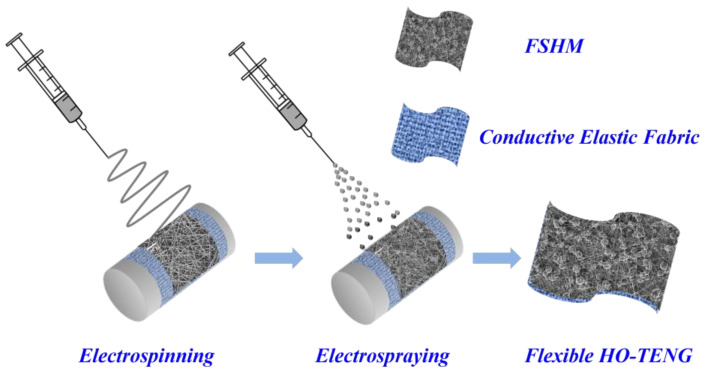
Flowchart for the flexible HO-TENG based on FSHM.

The HO-TENG was fabricated by electrospinning assisted with elecrospraying, and the conductive elastic fabric was used as the receiving substrate of FSHM and the electrode of HO-TENG, as presented in Scheme 1. The fabricated FSHM was served as the tribo-electronegative layer for the flexible HO-TENG, where a double-sided conductive copper tape was used to lead out the wire on the electrode. Finally, the size of the fabricated HO-TENG is 4.5 cm × 4.5 cm.

### 2.4. Characterization and Measurement

The experimental process of electrospinning and elecrospraying was carried out on a desktop electrospinning machine (nano apparatus, JDF05). The morphology of the fabricated nanofiber and electrospray microsphere was carried out using a field-emission scanning electron microscopy (ZEISS, SEM500, Jena, Germany) with an accelerating voltage of 10 kV, and the Nano Measurer 1.2 software was applied to measure the diameter of the nanofiber and the microsphere. Specifically, a number of fibers/microspheres (usually more than 100) are randomly counted in the Nano Measurer software (Nano Measurer 1.2)and the mean diameter and diameter distribution of the electrospun fibers/electrospray microspheres are obtained after systematic processing using the ruler on the SEM image as a standard. The thermal stability was performed on a TAQ600 thermogravimetric analyzer (TA Company, New Castle, DE, USA, 40–900 °C) at a rate of 10 °C min^−1^ under a nitrogen atmosphere. The electrical output characteristics (including the output voltage *V_oc_*, short-circuit current *I_sc_*, and transferred charge *Q_sc_*) of the TENG devices were measured by a high-impedance electrometer (6514 Keithley, Cleveland, OH, USA) at an operating frequency of 1.5 Hz.

## 3. Results and Discussions

The detailed fabrication process for the flexible HO-TENG in this study is schematically presented in Scheme 1. In brief, a series of single-electrode mode flexible HO-TENGs was established through electrospinning assisted with elecrospraying technology, in which PVDF-HFP has excellent tribo-electronegativity owing to its high electron affinity, large spontaneous polarization, and excellent polarization stability [48,49,50]. Therefore, PVDF-HFP is used as the triboelectric matrix material in this study, and the fabricated FM and FSHM were used as the tribo-electronegative layer, and the receiving matrix conductive fabric as the electrode. As it is well known that in the spinning process, the morphology and properties of electrospun fibers are affected by many factors [51,52,53] such as the distance between the emitter and the collector and the high voltage value between them, the solution concentration, the cylinder rotation speed, the needle diameter and the solution flow rate.

**Figure 1:**
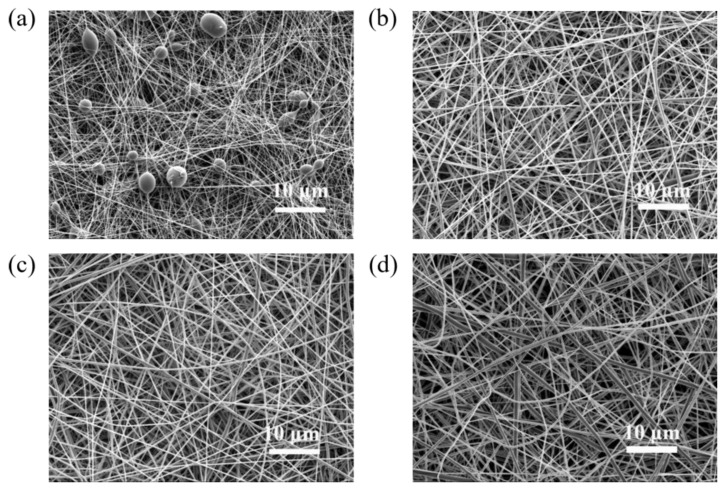
SEM micrographs of electrospun fibers prepared with different concentration spinning solutions. (**a**) 12 wt.%, (**b**) 20 wt.%, (**c**) 24 wt.%, (**d**) 28 wt.%.

**Figure 2:**
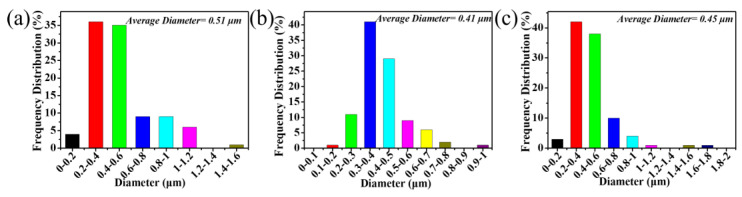
Diameters of electrospun fibers prepared with different concentration spinning solutions. (**a**) 20 wt.%, (**b**) 24 wt.%, (**c**) 28 wt.%.

In this study, the influence of the concentration of spinning solution on the morphology of electrospun fibers was investigated under the condition that other influencing factors were basically constant. First, to study the microstructure of the dielectric layer (tribo-electronegative layer) and explore the optimal concentration of the spinning solution during electrospinning, the surface morphology of the fabricated electrospun fibers was characterized by field-emission scanning electron microscopy and the corresponding results are shown in Figure 1. As presented in Figure 1, when the concentration of spinning solution is low (12 wt.%, Figure 1a) during the electrospinning process, the spinnability of the spinning solution is poor, and the collected nanofibers are uneven in thickness, small in diameter, and accompanied by a large number of spinning cones resulting in poor mechanical performance. With an increase in spinning solution concentration, the spinning cone gradually disappears, and the fiber morphology becomes regular. The corresponding average fiber diameters are displayed in Figure 2. It can be seen from Figure 1 and Figure 2 that when the spinning solution concentration is 24 wt.%, the fiber diameter is relatively uniform and the fibers in the FM prepared under this condition are closely stacked, which makes the fabricated FM have excellent mechanical properties [54,55]. Generally, good mechanical properties of electro-spun nanofiber membranes are positively related to their cycle stability (i.e., durability). Once the spinning solution concentration exceeds 24 wt.%, the diameter of the electro-spun nanofibers gradually becomes uneven, as displayed in Figure 1d and Figure 2c. This is because, compared with the low concentration spinning solution, the viscosity and surface tension of the high concentration spinning solution increases, and it is difficult for the droplets to form a jet, which can then easily cause a slight blockage at the needle tip, leading to a decrease in the jet flow rate, resulting in a large difference in fiber diameter. Therefore, 24 wt.% PVDF-HFP solution was selected as the spinning solution in the electrospinning process in the follow-up study.

**Figure 3:**
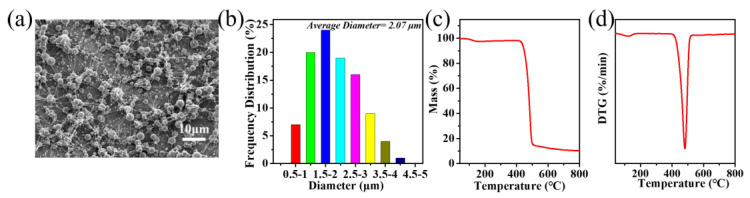
(**a**) SEM micrograph and (**b**) diameter distribution of electrostatic spray PVDF-HFP microspheres, (**c**) TGA and (**d**) DTG curve of FSHM.

Then, the surface morphology and average diameter distribution of PVDF-HFP microspheres prepared by elecrospraying technology were also characterized. As displayed in Figure 3a,b, the fabricated PVDF-HFP microspheres have different diameters, showing a regular spherical shape, and their average diameter is about 2.07 μm. In conclusion, the nanofibers and polymer microspheres with regular morphology were successfully prepared by electrospinning and elecrospraying technology in this study, which laid a foundation for the successful construction of the dielectric layer of nanofiber/microsphere hybrid film in the next step.

It is well known that the triboelectric skin, as the next-generation electronic device, can be used as the primary self-powerful interactive device for soft robots [56], human-machine interaction [57], and healthcare detection [58], which must be flexible, mechanically robust and environmentally stable for practical applications [20,59]. Much research has shown that electrospun FMs typically exhibit skin-like Young’s modulus, outstanding flexibility, and great stretchability [59,60,61], which is one of the reasons for choosing this preparation technology in this study. Furthermore, from Figure 3c,d, it can be seen that the fabricated FSHMs exhibit excellent thermal stability, environmental temperature stability, and their thermal resistance temperature is up to 438 °C, which is advantageous for FSHMs when considering their potential use as a energy harvester in harsh environments [62]. The weight loss in the range of 100 °C~140 °C is probably due to the volatility of residual solvents, i.e., DMF and acetone during electrospinning and elecrospraying [63]. Above all, the fabricated FSHMs can be widely used in the field of triboelectric devices for energy harvesting in harsh environments.

**Figure 4:**
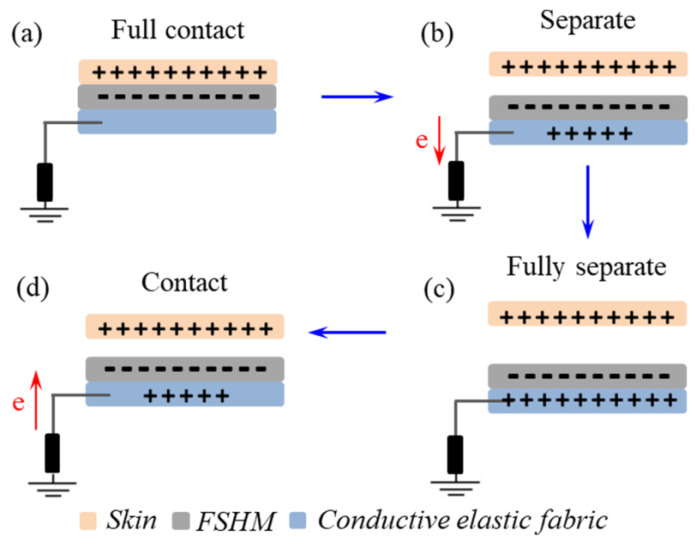
Working mechanism illustration of flexible HO-TENG.

As a sustainable energy harvester, TENGs can convert extensive existing low-frequency and irregular mechanical energy into valued electrical energy [64,65]. In this study, a series of single-electrode mode flexible HO-TENGs was established through electrospinning assisted with elecrospraying technology. Figure 4 illustrates the working mechanism of the fabricated HO-TENGs, where the CE and electrostatic induction were the basis for this working method [20,59,60,64]. When the external surface is in contact with the dielectric layer, electrification occurs due to the contact electrification and charge transfer between them. As seen in Figure 4a, the dielectric layer is negatively charged due to its high surface electron affinity. Once the two surfaces are gradually separated to a certain distance, a potential difference will be formed between the two surfaces, and then the negative charge on the dielectric layer will induce the electrode to generate a positive charge, and free electrons flow from the electrode to the ground, generating a pulse current (Figure 4b). When the external surface reaches the maximum displacement (full separation), the electrostatic balance is reached (Figure 4c). Afterwards, when the external surface moves again and gradually makes contact with the dielectric layer, the induced positive charge in the electrode gradually decreases (electrons gradually return to the electrode), and a reverse current signal is generated (Figure 4d). In the above continuous contact-separation process, the voltage and current pulses are constantly generated to convert mechanical energy into precious electrical energy.

**Figure 5:**
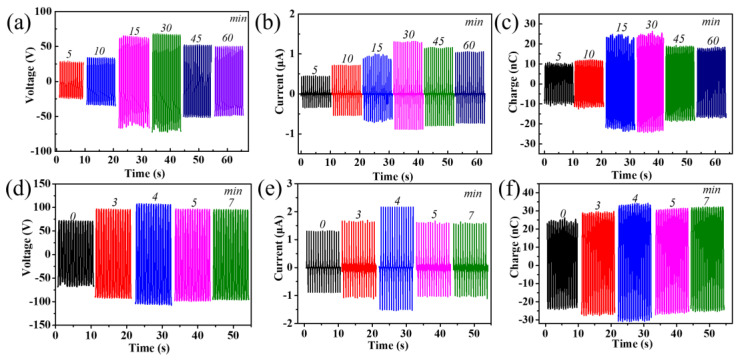
Electrical output of (**a**) *V_oc_*, (**b**) *I_sc_*, and (**c**) *Q_sc_* of the flexible HO-TENG based on FMs prepared at different electrospinning receiving times. Electrical output of (**d**) *V_oc_*, (**e**) *I_sc_*, and (**f**) *Q_sc_* of the flexible HO-TENG based on FSHMs prepared at different electrostatic spray receiving times.

To explore the electrical output performance of flexible HO-TENG, a series of electrical tests was carried out in this study. During the tests, the operating frequency and separation distance of the linear motor reciprocating motion are constant. First, to obtain the best collection time of FM in the electrospinning process, we tested and analyzed the electrical output of flexible HO-TENG based on FMs prepared at different electrospinning receiving times. As shown in Figure 5a,c, with the increase of receiving time, the electrical output of flexible HO-TENG shows an obvious trend of increasing first and then decreasing, and reaches the maximum value when the receiving time is 30 min. Therefore, to further improve the output performance of HO-TENG, the receiving time of FMs in the subsequent fabrication process of FSHM dielectric layer is fixed at 30 min. Furthermore, Figure 5d,e exhibits the electrical output performance of flexible HO-TENG based on FSHMs prepared at different electrostatic spray receiving times. As shown in these figures, the electrical output of HO-TENG based on FSHMs is higher than that of TENG based on single electrospun FM. This is mainly because the existence of electrospray microspheres increases the surface roughness of single electrospun FM, thus increasing the triboelectrification effect, which increases the triboelectrification charge [41]. In addition, the output performance (*V_oc_*, *I_sc_*, *Q_sc_*) of flexible HO-TENG based on FSHMs reached the maximum value of 212 V, 2.16 μA and 64 nC, respectively, when the electrospray time was 4 min. In summary, when the electrospinning and electrospray time are 30 min and 4 min respectively, the fabricated FSHM has the optimal triboelectric characteristics.

**Figure 6:**
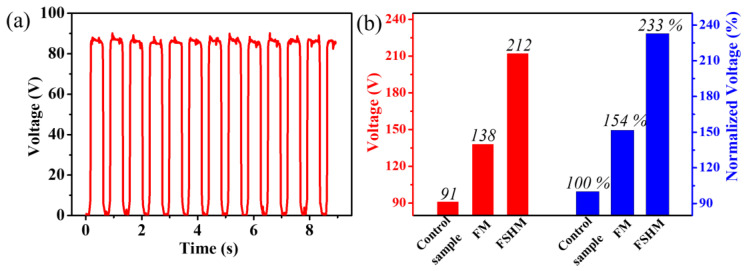
(**a**) *V_oc_* of flexible c-TENG, (**b**) comparison diagram of *V_oc_* of flexible TENG based on control PVDF-HFP membrane, FM and FSHM, respectively.

Additionally, the flexible TENG based on control PVDF-HFP membrane, which was prepared by blade coating technology, is introduced to further explain the improvement of HO-TENG output performance by the presence of micro/nanostructure on the dielectric layer surface. Figure 6a shows the *V_oc_* of the TENG based on control PVDF-HFP membrane (c-TENG), which is about 91 V. Moreover, the comparison diagram of *V_oc_* of flexible TENG based on control PVDF-HFP membrane, FM, and FSHM is shown in Figure 6b. From Figure 6b, the *V_oc_* of flexible HO-TENG based on FSHM and FM is 2.33 times and 1.54 times higher than that of c-TENG, respectively. The above results further confirmed that the output performance of TENG devices can be effectively improved by constructing micro/nanostructures on the contact interface of triboelectirc materials. The reason is that the existence of micro/nanostructures can increase the surface roughness of PVDF-HFP film, enhance the triboelectrification effect, and then increase the triboelectrification charge. It may also be that [66,67] (1) compared with the smooth plane structure, the contact between the external surface and the FSHM/FM dielectric layer is not a surface contact. There is an air layer in the middle, the existence of which can increase the effective dielectric constant, while the electrical output of TENG is positively correlated with the dielectric constant; (2) compared with the smooth plane structure, the tip discharge effect of micro/nano structures is more obvious, and triboelectric charges are easily generated.

**Figure 7:**
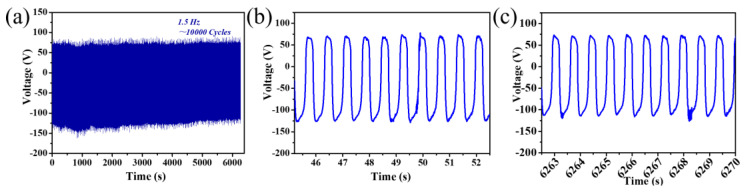
Long cycle stability and durability testing of flexible HO-TENG based on FSHM at 1.5 Hz motion frequency. (**a**) Stability and robustness measurement of flexible HO-TENG based on FSHM, (**b**,**c**) *V_oc_* at different times during the stability and robustness measurement of flexible HO-TENG based on FSHM.

In addition to the electrical output performance, the cyclic stability of TENG is also a key factor for its practical application. As displayed in Figure 7, the output voltage of flexible HO-TENG based FSHM does not show an obvious attenuation trend after 10,000 cycles of stability testing, which indicates that the flexible HO-TENG based FSHM prepared in this study has excellent cyclic stability and durability.

**Figure 8:**
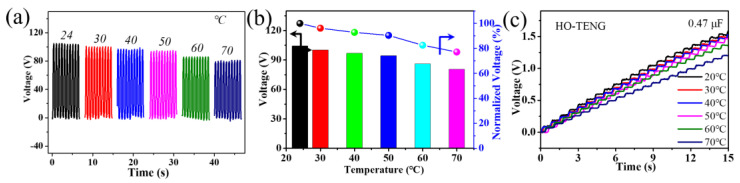
Electrical output of flexible HO-TENG at different operating temperature. (**a**) *V_oc_*, (**b**) normalized voltage, (**c**) charging performance of flexible HO-TENG to a commercial capacitor of 0.47 μF.

As is known to all, due to the influence of the thermionic emission effect in the process of CE, most TENGs cannot maintain an effective electrical output under high temperature conditions which shows obvious thermal negative effect and seriously affects the working stability and application range of TENG devices [28,29,32,33,34]. In recent years, to improve the working temperature of a TENG, scientists have carried out a series of related research and explorations. Although the TENG prepared by these studies can operate stably at a high temperature, its electrical output performance is significantly lower than that at room temperature [36,37,38]. For example, the output efficiency of tribological power generation devices based on PTFE/Nylon 66 [36], polystyrene/nylon [37] and PTFE/Cu [38] can only maintain 63.24% (70 °C), 26.82% (70 °C) and 51.87% (70 °C) at room temperature, respectively. In this study, a temperature-controlled hot stage was used as the heat source to simulate different temperature conditions during the operation of the HO-TENG device. A T-type thermocouple was used to measure the temperature of the HO-TENG device during operation, and the output performance of the HO-TENG in a series of high-temperature environments was explored. The corresponding results are shown in Figure 8a,b. From Figure 8a,b, the HO-TENG based on FSHM prepared in this study has a small output voltage attenuation and an output efficiency of more than 77% when working at a high-temperature environment of 70 °C. As listed in Table 1, compared with previous reports [36,37,39,44,68,69], the HO-TENG has a higher retention rate of electrical output performance in high-temperature environments and excellent high-temperature operability. In view of suppressing the electron thermionic emission of the triboelectric layer and improving the structural stability of triboelectric materials at high temperatures are the key to ensuring the efficient, stable and continuous operation of triboelectric materials in the changing environment, preparing the high-temperature operable TENG, and expanding its application fields. Through systematic literature research, we believe that the electrical output performance and operating temperature of high-temperature operable TENG devices can be further improved by adding high-temperature resistant dielectric filler to the triboelectric materials, using polymer materials with excellent thermal-resistance as the triboelectric layer, and and more refined micro/nanostructure design in subsequent studies [39,70,71], which is also the focus of our work in the future. In addition, as shown in Figure 8c, the HO-TENG prepared in this study shows excellent charging characteristics for commercial capacitors under different temperature environments, which provides unlimited possibilities for HO-TENG electronic devices to be applied in harsh environments.

## 4. Conclusions

In this study, without destroying its long-term durability, ease of manufacture and low cost, the FSHM tribo-electronegative layer with nanofiber/microsphere hybrid interface structure was successfully constructed by combining electrospinning and elecrospraying technology. This preparation technology has the advantages of simple preparation process and large-scale industrialization. The fabricated FSHMs exhibit excellent thermal stability and the thermal resistance temperature of them is up to 438 °C, which greatly promotes the application of TENG electronic devices in high-temperature environments. All HO-TENG devices based on FM and FSHM in this study present outstanding enhanced electrical output characteristics. Thereinto, the *V_oc_* of flexible TENG based on FSHM and FM is 212 V and 138 V, respectively, which is 2.33 times and 1.54 times higher than that of c-TENG. Additionally, the flexible HO-TENG shows excellent cyclic stability and durability. What is noteworthy is that this HO-TENG can maintain 77% of its output performance at 70 °C in comparison with room temperature, showing excellent high-temperature operability. Therefore, this study not only constructs TENG device with enhanced electrical output characteristics, which improves its practicability in modern intelligent devices (like autonomous vehicles), but also provides some experimental basis for effective collection of mechanical energy in high-temperature environments, and promotes the application of TENG devices in harsh environments (such as automobile engine room).

## Figures and Tables

**Table 1:** Normalized voltage/current (*χ*) of flexible HO-TENG in this study and those of previously reported similar studies.

Ref.	Triboelectric Pairs	Operating Temperature (°C)	*V_oc_*/*I_sc_*	*χ* (%)
[42]	OA-PS/Nylon	25	6.89 V	100.00
	OA-PS/Nylon	60	2.50 V	36.25
	OA-PS/Nylon	70	1.85 V	26.82
[36]	PTFE/Nylon 6.6	25	6.23 V	100.00
	PTFE/Nylon 6.6	70	3.94 V	63.24
[37]	PTEF/Cu	25	199.48 V	100.00
	PTEF/Cu	70	103.48 V	51.87
[69]	PTFE/Al	30	0.75 V	100.00
	PTFE/Al	40	0.30 V	40.00
	PTFE/Al	50	0.18 V	24.00
	PTFE/Al	60	0.16 V	21.33
[70]	Silicone/Skin	20	6.092 μA	100.00
	Silicone/Skin	60	4.437 μA	72.84
This study	FSHM/Cu	24	104.32 V	100.00
	FSHM/Cu	50	94.33 V	90.42
	FSHM/Cu	60	86.09 V	82.52
	FSHM/Cu	70	80.42 V	77.10

## Data Availability

Not applicable.
